# Estimating the Prevalence of Alzheimer's Disease Pathology Using Plasma Biomarkers in COMPASS‐ND

**DOI:** 10.1002/alz70856_103330

**Published:** 2025-12-25

**Authors:** Jennifer G Cooper, Nyra Ahmed, Johnny Huang, Bruna Seixas Lima, Durjoy Lahiri, David Yang, Sophie K Stukas, Mari L DeMarco, Howard Chertkow, Cheryl L Wellington

**Affiliations:** ^1^ University of British Columbia, Vancouver, BC, Canada; ^2^ University of Toronto, Toronto, ON, Canada; ^3^ Baycrest Academy for Research and Education, Toronto, ON, Canada; ^4^ Department of Medicine, Queen's University, Kingston, ON, Canada; ^5^ Baycrest Centre for Geriatric Care, Toronto, ON, Canada

## Abstract

**Background:**

The Comprehensive Assessment of Neurodegeneration and Dementia (COMPASS‐ND) study is a national Canadian observational study of participants clinically diagnosed with various forms of dementia and cognitive complaints. Here, we utilize plasma biomarkers including amyloid beta (Aβ42/40), phosphorylated tau‐181 (*p*‐tau‐181), neurofilament light (NfL), and glial fibrillary acidic protein (GFAP), to estimate the prevalence of Alzheimer's disease (AD) pathology in COMPASS‐ND. This enables us to assess concordance between clinical presentation and underlying pathogenesis in participants.

**Method:**

Biomarkers were analysed in COMPASS‐ND plasma samples (*n* = 936) using the Quanterix Simoa HD‐X analyzer with Neurology 4‐plex E and *p*‐tau‐181 assays. A sub‐cohort (*n* = 150) of participants with cerebrospinal fluid (CSF) Aβ42, *p*‐tau‐181 and total tau (Roche Elecsys assays) were utilized to determine plasma biomarker cut‐offs. Area under the receiver operating curve (AUROC) analysis was conducted. Cut‐offs, determined at the Youden's index, were then applied to the remaining COMPASS‐ND participants to estimate the percentage of biomarker positive individuals in each clinically determined diagnostic group.

**Result:**

Of the COMPASS‐ND participants with CSF, 80% (*n* = 120) were on the AD continuum. AUROC analysis revealed an area of 0.768 for Aβ42/40, 0.638 for *p*‐tau‐181, 0.559 for NfL, and 0.689 for GFAP. Cut‐offs for plasma biomarkers to be considered on the AD continuum were Aβ42/40 <0.068 pg/mL, *p*‐tau‐181 >2.7 pg/mL, NfL >18.6 pg/mL, and GFAP >134.9 pg/mL. A multi‐biomarker cut‐off based on having positive results for 2 out of 3 from Aβ42/40, *p*‐tau‐181, and GFAP, yielded a sensitivity of 64% and specificity of 83%. In the remaining *n* = 786 COMPASS‐ND participants, the multi‐marker panel estimated that AD pathology would be present in 30% (*n* = 28/93) of cognitively normal, 25% (*n* = 27/106) of subjective cognitive impairment, 52% (*n* = 167/321) of mild cognitive impairment, 83% (*n* = 96/116) of AD, 73% (*n* = 16/22) of Lewy body dementia, 45% (*n* = 13/29) of frontal temporal dementia, and 34% (*n* = 34/99) of Parkinson's disease participants.

**Conclusion:**

The plasma biomarkers assays tested have high specificity for detecting AD pathology, enabling their use to estimate AD pathology in a larger cohort. Based on the low sensitivity of the plasma biomarkers tested, we assume that these results underestimate the prevalence of AD pathology in the COMPASS‐ND cohort.

